# Serum level of hepatocyte growth factor is a novel marker of predicting the outcome and resistance to the treatment with trastuzumab in HER2-positive patients with metastatic gastric cancer

**DOI:** 10.18632/oncotarget.6753

**Published:** 2015-12-24

**Authors:** Naoki Takahashi, Koh Furuta, Hirokazu Taniguchi, Yusuke Sasaki, Hirokazu Shoji, Yoshitaka Honma, Satoru Iwasa, Natsuko Okita, Atsuo Takashima, Ken Kato, Tetsuya Hamaguchi, Yasuhiro Shimada, Yasuhide Yamada

**Affiliations:** ^1^ Division of Gastrointestinal Oncology, National Cancer Center Hospital, 5-1-1 Tsukiji, Chuo-ku, Tokyo, 104-0045, Japan; ^2^ Division of Clinical Laboratories, National Cancer Center Hospital, 5-1-1 Tsukiji, Chuo-ku, Tokyo, 104-0045, Japan; ^3^ Division of Pathology, National Cancer Center Hospital, 5-1-1 Tsukiji, Chuo-ku, Tokyo, 104-0045, Japan; ^4^ Division of Clinical Oncology, Kochi Health Sciences Center, 2125-1 Ike, Koch-city, Koch, 781–8555, Japan

**Keywords:** HER2, gastric cancer, trastuzumab, ligands, HGF

## Abstract

HER2-overexpression in tumor tissue is observed in 6 to 23% of advanced gastric cancer (GC) cases, and trastuzumab is an active molecular drug for these patients. There are no data available on whether serum levels of ligands are associated with the response and resistance to trastuzumab in HER2-positive patients with metastatic GC. HER2 screening of 502 patients with advanced gastric cancer was performed in our institution. Among these patients, 84 patients (16.8%) were diagnosed as HER2-positive, and those who were treated with trastuzumab and met the inclusion criteria were enrolled in the present study. Serum levels of ligands that affect the HER2 signal pathway were measured by an enzyme-linked immunosorbent assay. Forty-six HER2-positive patients were enrolled in this study, and 26 patients (56.5%) achieved a partial response to treatment with trastuzumab. Among several ligands, the serum level of hepatocyte growth factor (HGF) was significantly lower in responders compared with that in non-responders (*p* = 0.014). Multivariate analyses showed that a high level of serum HGF was a poor prognostic factor for overall survival (OS) compared with low levels of HGF (adjusted HR: 3.857, 95% CI: 1.309–11.361, *p* = 0.014). Among 25 patients without initial disease progression on the treatment with trastuzumab, the mean value of serum HGF at disease progression was significantly higher than that at pre-treatment (*p* = 0.041). As novel findings, our study indicated that serum level of HGF was associated with tumor shrinkage and time to progression of trastuzumab in HER2-positive patients with metastatic GC.

## INTRODUCTION

Gastric cancer (GC) is a common cancer and a leading cause of death from cancer in the East Asian countries [1]. Survival of patients with metastatic GC has been moderately improved because new molecular target drugs have been developed during the last decades. On the other hand, many patients are often diagnosed at an advanced stage which there is a poor prognosis due to the high frequency of recurrence and distant metastasis [2].

Human epidermal growth factor receptor-2 (HER2) belongs to the human epidermal growth factor receptor family encoded by a gene located on the long arm of chromosome 17 [3]. The frequency of HER2 amplification in patients with gastric cancer was previously reported as 6% to 23% [4–6]. Several retrospective studies have reported that HER2 positivity is a prognostic factor associated with increased risk of invasion, metastasis, and worse survival [7–9]. Trastuzumab is a humanized monoclonal antibody that induces antibody-dependent cellular cytotoxicity and inhibits HER2-mediated signaling and prevents cleavage of the extracellular domain of HER2. The ToGA trial revealed that addition of trastuzumab to a fluoropyrimidine plus cisplatin regimen as first-line chemotherapy improved outcomes compared with chemotherapy alone in HER2-positive patients with gastric or gastro-esophageal junction (GEJ) adenocarcinoma [10]. Furthermore, phase II trials of oral S-1 plus cisplatin with trastuzumab as first-line chemotherapy and a combination of paclitaxel plus trastuzumab as second-line chemotherapy in trastuzumab naïve patients with HER2-positive metastatic GC were performed in Japan [11, 12]. These studies showed promising outcomes and well-tolerable profiles. International phase III trials of HER2-target agents such as pertuzumab and trastuzumab emtansine for HER2-positive patients with advanced gastric or GEJ cancer are now in progress [13].

Ligands of transmembrane receptor tyrsine kinases play important roles in cell proliferation, survival, migration and differentiation in tumor cells. Ligands of the HER family in humans comprise epidermal growth factor (EGF), transforming growth factor-alpha (TGF-alpha), heparin binding-EGF (HB-EGF), betacellulin, amphiregulin (AREG), epiregulin (EREG), epigen and neuregulin (NRG) [14]. Other than these ligands of the HER family, up-regulation of the HGF/MET pathways has been described as potential mechanisms of signal escape in solid tumors during molecular-target therapy of EGFR or HER2 [15–18]. In breast cancer, activation of HGF/MET/PI3K pathway drives tumor formation, metastasis, and drug resistance because of the crosstalk between MET and other oncogenic pathways [19]. Previous report indicated that HGF level is associated with epithelial to mesenchymal transition in patients with small cell lung cancer (SCLC) and high level of serum HGF were associated with low response to treatment and poor prognosis in stage IV SCLC patients [20]. In addition, increasing signaling of the insulin growth factor-1 (IGF-1)/IGF-1 receptor (IGF-1R) pathway was previously reported as one of the mechanism of resistance to trastuzumab in HER2-postive tumors [21–23]. Few reports have investigated the role of ligands as biomarkers in the treatment with trastuzumab in patients with metastatic GC. In the present study, we evaluated the roles of serum ligands in predicting the efficacy of and acquired resistance to trastuzumab in HER2-positive patients with metastatic GC.

## RESULTS

A total of 46 patients met the inclusion criteria between March 2010 and July 2014. Background characteristics are summarized in Table 1. The median age was 68 years, and most patients were male (93.5%) with PS 0–1 (95.3%). The histological tumor types comprised the intestinal (71.7%) and diffuse types (28.3%). The HER2 status comprised IHC 3+ (84.8%) and IHC2+/FISH (15.2%). Thirty-one patients received some subsequent treatment after failure of trastuzumab. The median number of cycles of trastuzumab treatment was 6 (range: 2–28). Six patients continued to receive the treatment with trastuzumab at the time of analysis of the present study.

**Table 1:** Patients' characteristics

Patients' characteristics	*N* = 46
**Median Age (range)**	68 (36–85)
**Gender (%)**	
Male	43 (93.5)
Female	3 (6.5)
**ECOG PS (%)**	
0	9 (19.6)
1	35 (76.1)
2	2 (4.3)
**Stage**	
Stage IV	29 (63.0)
Recurrence	17 (37.0)
**Histological type (%)**	
Differentiated adenocarcinoma (pap, tub)	33 (71.7)
Undifferentiated carcinoma (por, sig, muc)	13 (28.3)
**Primary site (%)**	
Proximal	22 (47.8)
Distal	24 (52.2)
**Number of metastatic site (%)**	
1	21 (45.7)
2 ≤	25 (54.3)
**Metastatic site (%)**	
Liver	27 (56.5)
Lung	6 (13.0)
Peritoneum	13 (28.3)
Lymph node	32 (69.6)
**HER2 status (%)**	
IHC 3+	39 (84.8)
IHC 2+ / amplification +	7 (15.2)
**Treatment line of trastuzumab (%)**	
1st	25 (54.3)
2nd to 3rd	21 (45.7)
**Treatment regimen with trastuzumab (%)**	
Fluoropyrimidine plus cisplatin	24 (52.2)
S-1	2 (4.3)
Paclitaxel	20 (43.5)
**Subsequent treatment (%)**	
Yes	31 (67.4)
No	15 (32.6)

Abbreviations: ECOG = Eastern Cooperative Oncology Group, PS = performance status, pap = papillary adenocarcinoma, tub = tubular adenocarcinoma, por = poorly differentiated adenocarcinoma, sig = signet cell adenocarcinoma, muc = mucinous adenocarcinoma, IHC = immunohistochemistry.

**Figure 1:**
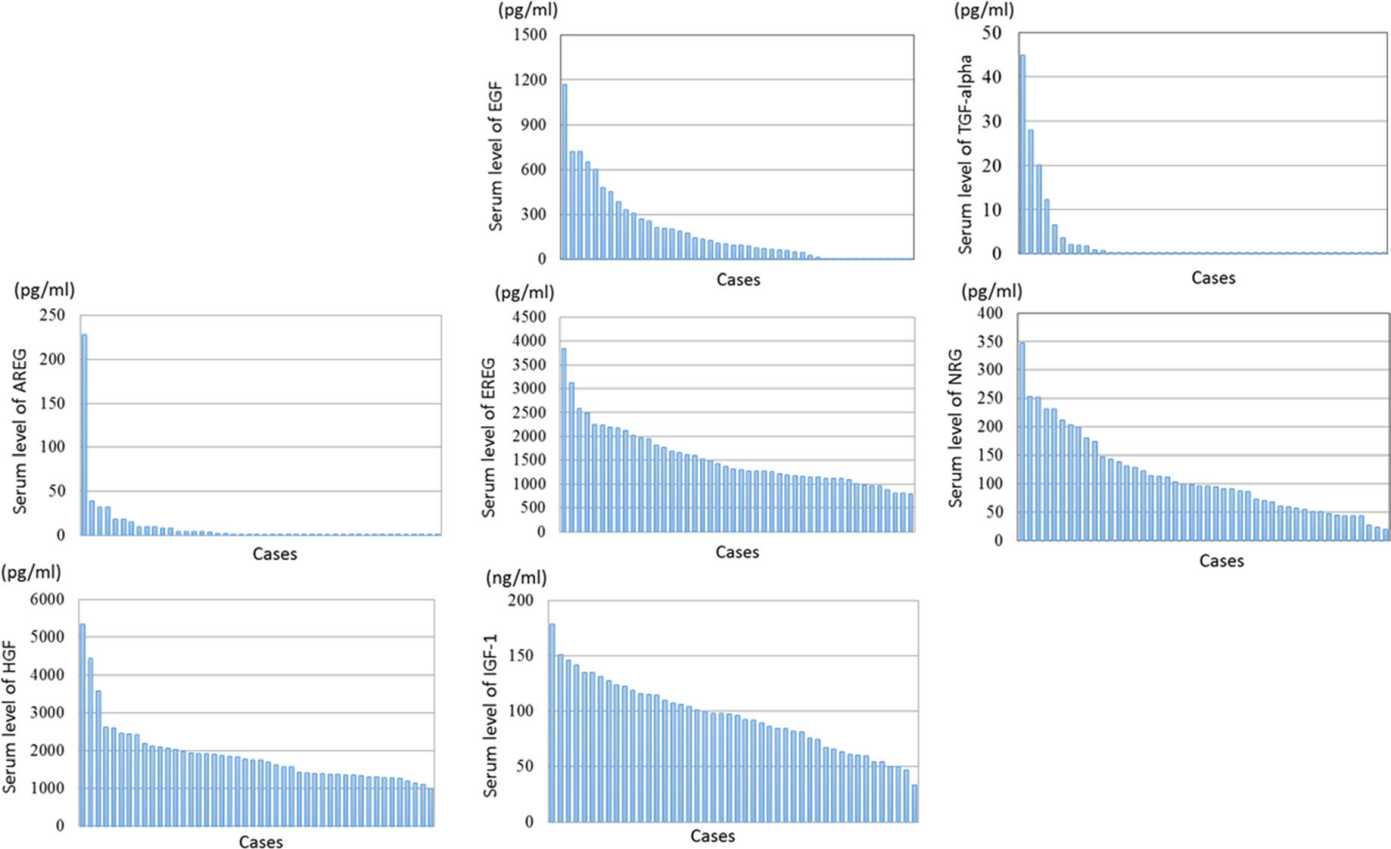
Graphic representations of serum levels of ligands by ELISA Serum levels of EGF, TGF-alpha, EREG, AREG, NRG, HGF and IGF-1 are shown in Table 1 The serum levels of some samples of EGF (*n =* 8), TGF-alpha (*n =* 35) and AREG (*n =* 26) were less than the lower limits of the detectable range of ELISA.

Serum samples from 46 patients were used to measure the concentrations of ligands. The pre-treatment serum levels of ligands are shown by graphical representations in Figure 1. Serum levels of EREG, NRG, HGF and IGF-1 were within detectable range of ELISA for all patients. On the other hand, serum levels of some samples of EGF (*n =* 8), TGF-alpha (*n =* 35) and AREG (*n =* 26) were less than the lower limit of the detectable range of ELISA. The median serum levels of EGF, EREG, NRG, HGF and IGF-1 were 94.9 pg/ml (range: < 0.001 – 1167 pg/ml), 1,307.3 pg/ml (791.2 – 3841.3 pg/ml), 95.9 ng/ml (20.1 – 347.6 ng/ml), 1,747.1 pg/ml (984.9 – 5340.7 pg/ml) and 96.6 ng/ml (33.1 – 178.6 ng/ml), respectively. For evaluation of the potential prognostic roles of these ligands, cut-off values to divide into high and low levels of serum ligands were determined as the median value for each ligand. Serum levels of TGF-alpha (range: < 0.001 – 44.86 pg/ml) and AREG (< 0.001 – 227.8 pg/ml) in more than half of patients were less than the lower limits of the detectable range. Therefore, cut-off values of serum TGF-alpha and AREG were determined as the lower limits of the measurable range.

### Comparison of serum levels of ligands between responders and non-responders

**Figure 2:**
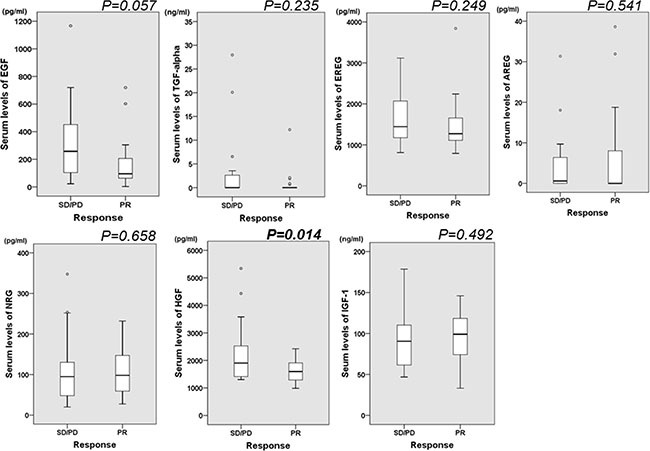
Box-and-Whisker plot illustrating the spread of data between serum ligands and the response to the treatment with trastuzumab The width of each box plot is drawn proportional to the square root of the number of observations in the groups. The serum level of HGF was lower in responders compared with non-responders. Among other ligands, there was no significant difference in serum levels of ligands between responders and non-responders.

Among 46 of HER2-positive patients who were enrolled in this study, 26 patients (56.5%) achieved PR by the treatment with trastuzumab and these patients were defined as responders. On the other hand, 12 patients (26.1%) achieved SD, and 8 patients (17.4%) were diagnosed as PD in the treatment with trastuzumab. These patients were defined as non-responders. We compared the serum levels of ligands between responders and non-responders, and the results are shown in Figure 2. Among several ligands, serum levels of HGF were significantly lower in responders compared with non-responders (*p =* 0.014). On the other hand, there were no significant differences statistically in serum levels of other ligands between responders and non-responders.

### Prognostic analyses of survival in HER2-positive patients treated with trastuzumab

Univariate and multivariate analyses of prognosis in terms of OS are shown in Table 2. Univariate analyses of our study showed that peritoneal dissemination (HR: 3.926, 95%CI: 1.417–10.787, *p =* 0.008), high EREG (HR: 3.413, 95%CI: 1.264–9.214, *p =* 0.015) and high HGF (HR: 3.894, 95%CI: 1.417–10.459, *p =* 0.008) were significantly associated with worse OS. Other serum ligands were not significantly associated with survival of HER2-positive patients treated with trastuzumab. Multivariate analyses showed that peritoneal dissemination (HR: 1.815, 95%CI: 1.172–3.053, *p =* 0.025) and high HGF (HR: 3.857, 95%CI: 1.309–11.361, *p =* 0.014) were significant prognostic factors in OS.

**Table 2:** Prognostic analyses of overall survival (OS) in HER2-positive patients treated with trastuzumab

	OS					
	Univariate analysis			Multivariate analysis		
	HR	95% CI	*P*-value	HR	95% CI	*P*-value
**ECOG PS**						
0	1					
1–2	2.598	0.749 – 9.007	0.132			
**Gender**						
male	1					
female	0.731	0.168 – 3.191	0.677			
**Age**						
≤ median	1					
> median	1.150	0.456 – 2.898	0.767			
**Primary site**						
proximal	1					
distal	0.989	0.400 – 2.441	0.980			
**Stage**						
stage IV	1					
recurrence	1.517	0.594 – 3.871	0.381			
**Histological type**						
diffuse type	1					
Intestinal type	0.498	0.831 – 3.705	0.172			
**HER2 status**						
IHC2 + /ISH+	1					
IHC3 +	0.701	0.202 – 2.440	0.557			
**Prior gastrectomy**						
yes	1					
no	0.617	0.317 – 1.977	0.792			
**Metastatic site**						
1	1					
2 or more	1.715	0.672 – 4.381	0.259			
**Peritoneal dissemination**						
no	1			1		
yes	**3.926**	1.429 – 10.787	**0.008**	**1.815**	1.172–3.053	**0.025**
**Liver metastasis**						
no	1					
yes	1.038	0.413 – 2.613	0.936			
**Treatment**						
F+C+Tmab (First-line)	1			1		
PTX+Tmab (Second/third-line)	1.591	0.642 – 3.942	0.316	1.515	0.728 – 3.153	0.115
**Serum EGF levels**						
low	1					
high	0.785	0.226 – 2.722	0.703			
**Serum TGF-alpha levels**						
low	1					
high	1.551	0.546 – 4.405	0.410			
**Serum AREG levels**						
low	1					
high	1.726	0.667 – 4.469	0.261			
**Serum EREG levels**						
low	1			1		
high	**3.413**	1.264 – 9.214	**0.015**	1.968	0.651 – 5.946	0.230
**Serum HGF levels**						
low	1			1		
high	**3.849**	1.417 – 10.459	**0.008**	**3.857**	1.309 – 11.361	**0.014**
**Serum NRG levels**						
low	1					
high	0.706	0.327 – 2.113	0.835			
**Serum IGF-1 levels**						
low	1					
high	0.579	0.223– 1.505	0.262			

Abbreviations: ECOG PS; Eastern Cooperative Oncology Group Performance Status, PFS; progression free survival, CI; confidence interval, F + C + Tmab; fluoropyrimidine/cisplatin/trastuzumab, PTX+ Tmab; paclitaxel/trastuzumab, EGF; epidermal growth factor, AREG; amphiregulin, EREG; epiregulin, HGF; hepatocyte growth factor, NRG; neuregulin, IGF-1; insulin growth factor-1.

A comparison of patients' backgrounds and histological findings between subgroups of high/low levels of serum HGF and EREG are shown in Supplementary Material 3. There were no significant differences in patient characteristics between subgroups of high/low levels of serum HGF, but liver metastasis was frequently observed in patients with high levels of serum HGF compared to those with low levels of HGF. In terms of EREG, there were no significant differences in patient characteristics regardless of serum level of EREG, but peritoneal dissemination was more frequently observed in patients with high levels of serum EREG compared to those with low EREG.

### Optimal cut-off values of serum HGF and survival curves for each chemotherapeutic regimen

**Figure 3:**
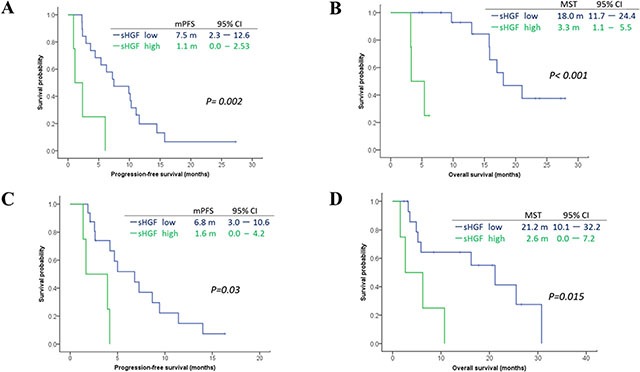
Survival curves according to serum levels of HGF (optimal cut-off level: 2,422.3 pg/ml) in subgroup treated with a combination of fluoropyrimidine plus cisplatin with trastuzumab (A–B) and weekly paclitaxel with trastuzumab (C–D) We evaluated the optimal cut-off value (2,433.3 pg/ml) of serum HGF by ROC curves analyses Among patients who received fluoropyrimidine plus cisplatin with trastuzumab as first-line chemotherapy, there were significant difference in PFS and Overall survival (OS) between high/low HGF groups (A–B). Among patients who received paclitaxel with trastuzumab, there was also significant differences in PFS and OS between high/low HGF groups (C–D).

We evaluated the cut-off value for serum HGF in order to set it so that it became an ORR best by an ROC curves analysis. As a result, the optimal cut-off value for serum HGF was set at 2,422.3 pg/ml, and we divided the patients into two groups, those with high HGF (*n =* 8) and those with low HGF (*n =* 38). There were significant better ORR in patients with low HGF compared to those with high HGF (ORR: 65.8 vs 12.5%, *p =* 0.014). In the present study, combinations of fluoropyrimidine and cisplatin with trastuzumab as first-line chemotherapy and paclitaxel with trastuzumab as second/third-line chemotherapy were the main chemotherapeutic regimens. Among patients who received fluoropyrimidine plus cisplatin with trastuzumab, those with high levels of serum HGF had significantly shorter PFS (median PFS: 1.1 months vs 7.5 months, *p =* 0.002) and median OS (3.3 months vs 18.0 months, *p* < 0.001) compared to those with low HGF. Among patients receiving paclitaxel with trastuzumab, those with high levels of serum HGF had significantly shorter PFS (median PFS: 1.6 months vs 6.8 months, *p =* 0.03) and median OS (21.2 months vs 2.6 months, *p =* 0.015) compared to those with low HGF. These results are shown in Figure 3.

### Change of serum HGF levels from pre-treatment to progressive disease by the treatment with trastuzumab

**Figure 4:**
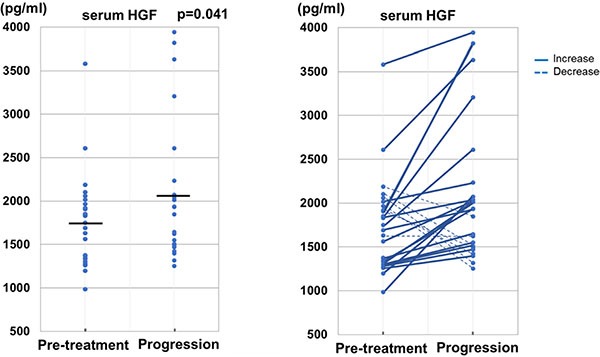
Change of serum level of HGF from pre-treatment to disease progression on the treatment of trastuzumab in patients without initial disease progression The mean value of serum HGF at disease progression was significantly higher than that at pre-treatment (*p =* 0.041). Elevation of serum HGF from pre-treatment to disease progression were observed in about three-quarters of patients (72%).

We could obtain 30 serum samples at disease progression during the treatment with trastuzumab from Biobank in our institution. We investigated the change of serum HGF from pre-treatment level to the level at progressive disease during the treatment in order to evaluate the role of serum HGF in developing resistance to trastuzumab. Among 25 patients who achieved PR or SD as the initial response to treatment with trastuzumab, the mean value of serum HGF at progressive disease was significantly higher than that at pre-treatment (2038.7 ± 793.6 pg/ml vs 1758.8 ± 536.9 pg/ml, *p =* 0.041). In addition, serum levels of HGF at progressive disease were elevated from the pre-treatment levels in 72% of HER2-positive patients. These results are shown in Figure 4.

### Association between serum levels of HGF at pre-treatment of first-line chemotherapy and immunohistochemistry of HER2

Additionally, 61 FFPE tumor sections of HER2-negative (*n =* 50) and HER2-positive (*n =* 11) metastatic GC patients who received first-line chemotherapy were collected and HER2 staining were evaluated. Matched-pair serum samples were also collected and serum level of HGF were measured by same ELISA kit. Serum levels of HGF by HER2 expression were summarized in Supplementary Data 4. The median of serum HGF by HER2 expression were 1,089.6 pg/ml (IHC0, *n =* 23), 1,055.4 pg/ml (IHC1+, *n =* 12), 1,157.9 pg/ml (IHC2+, *n =* 22), 1,303.6pg/ml (IHC3+, *n =* 30), respectively. The stronger HER2 expression of tumor tissues became, the higher serum level of HGF were detected in patients who received first-line chemotherapy (*p* = 0.027, Kruskal Wallis test).

### Additional analyses of serum HGF at pre-treatment of first-line chemotherapy with trastuzumab in HER2-positive patients

As additional cohort, eleven HER2-positive patients who were treated with fluoropyrimidine plus cisplatin with trastuzumab as first-line treatment were enrolled to our study. Median of serum HGF in these patients were 1,059.4 pg/ml (range: 717.5–1,657.4). Seven patients achieved PR and two patients achieved PD as best of response. Serum samples of additional cohort were collected from Biobank and analyzed serum levels of HGF. Among these patients, serum levels of HGF before treatment in patients who achieved PR (mean: 979.7 pg/ml, *n =* 7) were lower than those who achieved PD (mean: 1,634.0 pg/ml, *n =* 2).

## DISCUSSION

We investigated whether serum levels of ligands are associated with the efficacy of chemotherapy plus trastuzumab in HER2-positive patients with metastatic GC. As novel findings, a low level of serum HGF at pretreatment was significantly associated with a positive response to the treatment and a high level of serum HGF was a poor prognostic factor on the treatment with trastuzumab. At the failure of trastuzumab, serum level of HGF was frequently elevated compared with that at pretreatment. Other ligands were not significantly associated with the response to the treatment with trastuzumab or with the prognosis for the disease.

The mechanism of resistance to trastuzumab and anti-HER2 tyrosine kinase inhibitor (TKI) have been mainly investigated in breast cancer. Resistance to anti-HER2 treatment is caused by genetic or environmental alterations of receptors tyrosine kinase (RTKs) and downstream signals such as enzymatic cleavage of HER2 extradomain (ECD), constitutive activation of p95HER2, genetic alteration of the PI3K/AKT pathway and MARK pathway, PTEN loss, and *FcγRIIIa* polymorphisms as immune mechanism of antibody-dependent cytotoxicity [25–27]. To our knowledge, no reports have been published that evaluate the clinical roles of serum levels of ligands that activate the ErbB family, cMET and IGF-1R pathways on the treatment with trastuzumab in HER2-positive patients with metastatic GC. In metastatic colorectal cancer, we previously investigated the clinical role of serum ligands in predicting the prognosis of anti-EGFR antibody treatment [28]. Similar to the result of the present study, high levels of serum HGF was associated with poor prognosis in *KRAS* wild-type patients with metastatic colorectal cancer. This indicated that serum HGF may be a potent marker to predict resistance to molecular-target agents that inhibit the receptors tyrosine kinase (RTKs) of the ErbB-family in gastrointestinal cancers.

In the present study, we also evaluated the change of the serum level of HGF at trastuzumab failure during treatment to evaluate the role of serum HGF in inducing acquired resistance to trastuzumab. The mean value of the serum level of HGF at disease progression was significantly higher than that at pre-treatment in metastatic GC patients who were treated with trastuzumab. As previous reports, Kyoury H *et al.* [29] indicated that physiological signals downstream from the HGF/Met receptor synergize with ErbB2/Neu to enhance the malignant phenotype, promoting the breakdown of cell-cell junctions and enhanced cell invasion. Overexpression and increased gene copy numbers of HGF and MET in tumor tissues were associated with the resistance to and failure of trastuzumab treatment in HER2-postive breast cancer [30, 31]. These findings support that there is cross-talk of signal pathways between members of the ErbB family and HGF/Met pathway on the treatment with trastuzumab.

In our study, serum levels of HGF were different by HER2 expression of gastric cancer tissues. This indicated that activation of HGF/MET pathway may be different between HER2-positive and HER2-negative patients. Zhang *et al.* identified that some RTKs pathway including HGF/MET pathway caused unresponsiveness to HER2 inhibitor (lapatinib) by pre-clinical model of gastric cancer [32]. In this study, adding HGF to lapatinib treatment in MET-overexpressed HER2 gastric cancer cells not only activated MET, but also led to restimulation of downstream effectors AKT and ERK1/2 even in the presence of lapatinib, although phosphorylation of HER2 and EGFR continued to be suppressed. Furthermore, addition of MET inhibitor to treatment of lapatinib plus HGF inhibited HGF-induced MET phosphorylation and AKT, ERK1/2 activation. These findings demonstrated that MET activation confers lapatinib resistance through restimulating the MAPK and AKT signaling pathways in MET/HER2 overexpressed gastric cancer cells. In addition, MET/p-MET expression was significantly observed in HER2-positive gastric cancer tissues. Taken together, activation of HGF/MET pathway were strongly associated with the resistance to HER2 inhibitor and this mechanism were more frequently observed in HER2-positve gastric cancer patients compared with HER2 negative patients.

There is few report that described the relationship between serum HGF and HER2 in gastric cancer. In preclinical study, previous report indicated that HGF activation of MET receptors rescued HER2-amplifed gastric cancer cells from lapatinib-induced growth inhibition by re-stimulating the downstream pathways and restoring normal cell-cycle progression [33]. A report has been published that evaluated the clinical roles of serum HGF as a prognostic factor in advanced GC. Park do J *et al.* measured serum levels of VEGF-A, HGF, fibroblast growth factor 2 (FGF2) and epidermal growth factor (EGF) and evaluated the prognostic role of these ligands in advanced GC patients who underwent gastrectomy [34]. In this study, patients with high level of HGF had shorter survival compared with those with low HGF by a log-rank test, and serum HGF also was had positively correlated with disease progression, stage and tumor metastasis. Similar to overexpression or gene amplification of MET, these findings support our results that HGF induce the resistance to HER2-target drug in HER2-posotive gastric cancer patients.

In locally advanced gastric cancer, the prognostic values of HGF/MET expression in tumor tissue has also been investigated and found to be a poor prognostic factor in patients who were treated by surgical resection or chemotherapy [35–41]. The prevalence of amplification of the MET gene was rare (less than 5%), and overexpression (more than IHC2+) of MET protein in tumor tissues were observed by IHC in 9–20% of advanced GC patients who previously underwent gastrectomy. High expressions of mRNA of HGF and MET in tumor tissues were also associated with poor prognosis in advanced GC patients who underwent gastrectomy [38]. It was concluded from these studies that activation of the HGF/MET pathway in tumor tissue plays significant roles in tumor progression and prognosis of locally advanced GC.

Prognostic value of HER2 status in metastatic GC patients was previously investigated worldwide. In Western countries, previous reports evaluated the prognostic role of HER2 status in patients who were enrolled in first-line prospective trials [42]. HER2-positive patients had longer survival compared with HER2-negative patients, but multivariate analyses showed that HER2 was not independently prognostic factor. In our institution, prognostic role of HER2 status in metastatic GC patients was previously evaluated [43, 44]. There was no significant difference in OS between HER2-positve patients and HER2-negative patients. These results suggested that HER2 status was not a prognostic factor in metastatic GC patients.

Therapeutic agents of the HGF/MET pathway, anti-HGF or anti-MET antibodies such as rilotumumab and onartuzumab have been developed for use in unresectable GC patients. As potential predictive markers of molecular-target therapy involving the HGF/MET pathway, amplification and copy number of the MET gene, mRNA expression of MET and HGF, and immunohistochemistry (IHC) of HGF/c-MET have been investigated in GC. In a randomized placebo-controlled phase II study, the efficacy of the anti-HGF antibody rilotumumab was evaluated in combination with platinum-based chemotherapy as first-line treatment in patients with advanced gastric or GEJ adenocarcinoma [45]. The combination of rilotumumab plus chemotherapy marginally improved both PFS and OS in the intention-to-treat population. A MET-positive subgroup (IHC ≥ 1+) achieved longer OS compared with MET-negative patients by chemotherapy plus rilotumumab (HR 0.29, *p =* 0.012). In contrast, there was no clinical benefit of adding rilotumumab to chemotherapy in MET-negative patients. According to this study, MET-positivity by IHC in tumor tissues is considered a predictive marker for efficacy of anti-HGF antibody in metastatic GC patients. Recently, two large-scale randomized phase III trials (RILOMET-1 study: rilotumumab, MetGastric study: onarutuzumab) evaluated the efficacy of adding HGF/MET inhibitor to first-line chemotherapy in MET-positive patients with unresectable gastric or GEJ adenocarcinoma [46, 47]. Unfortunately, these drugs did not provide additional efficacy to first-line chemotherapy in these phase III trials. In a search for an explanation of the negative results of HGF/MET inhibitors, it was considered whether MET expression by IHC is a true indicator of the efficacy of MET/HGF inhibitors and whether there is heterogeneity of MET expression in GC tissues. Biomarker analyses, including the MET expression in these phase III trials, will clarify the role of MET expression by IHC, and further research on other predictive markers of HGF/MET inhibitors, including the serum level of HGF, are required in advanced GC patients. In addition, positive effect of low dose of rapamycin on survival of caner-prone HER-2/neu mice have been reported [48]. Strategy to use rapamycin in combination with anticancer drugs may be better for HER2-overespressed patients with activation of MET/PI3K pathway and mTOR signaling.

There is some limitations in the present study. First, we excluded some unresectable HER2-positive patients who were treated with trastuzumab. For example, those whose serum samples were not stocked for performing the ELISA and those without some tumor target lesions and could not be evaluated for a response to the treatment by RECIST criteria. Second, the sample size of HER2-positive patients in the present study is small, so further evaluation in larger-scale analyses of prospective study are needed to confirm whether our findings are reproducible.

In conclusion, this is the first analysis to evaluate the clinical roles of serum ligands in the treatment with trastuzumab in HER2-positive patients with metastatic GC. The serum level of HGF at pre-treatment is a potent biomarker to predict the initial response to treatment and prognosis on the treatment with trastuzumab in HER2-positive GC patients. In addition, elevation of the serum level of HGF at disease progression was observed as acquired resistance in about three-quarters of patients without early failure of trastuzumab. Time-dependent changes of serum HGF during the treatment with trastuzumab may provide significant information in terms of an individual doctor's decisions to select the most appropriate treatment as personalized medicine and the serum markers are less invasive compared with the biopsy or surgical resection of tumor tissues for metastatic stage of GC patients. Further larger-scale analyses of prospective study are required to validate our findings of the present study.

## MATERIALS AND METHODS

### Patients and sample collection

Between August 2008 and August 2014, HER2 screening was performed on formalin-fixed, paraffin-embedded samples from endoscopic biopsy or surgical resection from 504 patients with advanced gastric cancer at the Gastrointestinal Oncology Division in National Cancer Center Hospital, Tokyo. Among 504 patients who underwent HER2 screening, 84 patients (16.7%) were diagnosed as HER2-positive. Finally, we enrolled 46 HER2-positive patients in this study, and the inclusion criteria was as follows: Histologically confirmed as adenocarcinoma of gastric or GEJ cancer, positive HER2 status, unresectable stage (stage IV or recurrence), received treatment with trastuzumab (at least 2 cycles), age ≥20 years Eastern Cooperative Oncology Group (ECOG) performance status (PS) 0–2, adequate hematological and organ functions, at least one measurable target lesion, no metastasis to brain or central nervous system, no concomitant cancer except for gastric or GEJ adenocarcinoma, sufficient serum samples at pre-treatment and patient's consent to the use of clinical data and materials. A summarizing diagram of the present study is presented in Supplementary Material 1.

Blood samples in our study were obtained from residual blood samples of previous laboratory tests. Separated serum samples were immediately stored at −20°C in the Biobank of the division of clinical laboratories in the National Cancer Center Hospital until use. We collected serum samples from these patients within a month before initiation of the treatment with trastuzumab. In addition, serum samples were collected from 30 of 46 patients during progression of their disease during trastuzumab treatment. Patients continued to receive the treatment until disease progression or intolerable toxicity from chemotherapy intervened. The response to the treatment was evaluated by contrast-enhanced CT every 2 to 3 months. This study was undertaken after approval by the institutional review boards.

### Treatment with trastuzumab

All patients received trastuzumab in combination with cytotoxic agents. Trastuzumab was administered at 6 mg/kg on the first day, followed by 4 mg/kg every 3 weeks. Chemotherapeutic regimens with trastuzumab were fluoropyrimidine plus cisplatin (*n =* 24, 52.2%), monotherapy of S-1 (*n =* 2, 4.3%), paclitaxel (*n =* 20, 43.5%). Trastuzumab was administered in combination with the following chemotherapy regimens: Fluoropyrimidine plus cisplatin consisted of capecitabine plus cisplatin (*n =* 15), S-1 plus cisplatin (*n =* 7) and 5-FU plus cisplatin (*n =* 2). Twenty-five (54.3%) patients were treated as first-line chemotherapy, and 21 patients (45.7%) were treated as second- or third-line chemotherapy. Schedules and doses of cytotoxic agents of the chemotherapeutic regimens were referred to in previous reports of clinical trials [4, 11, 12]. Dose reduction or drug withdrawal was appropriately performed according to each doctor's decision. There were no restrictions of subsequent treatment after failure of the treatment with trastuzumab.

### Immunochemistry and dual color *in situ* hybridization (DISH) assay of HER2

Tumor specimens were taken by endoscopic biopsy or from resected primary lesions and tested for HER2 status with IHC (Hercep Test, Dako, Denmark); expression of HER2 was evaluated using the criteria as described in a previous report [24]. Pathological diagnosis was confirmed by two pathologists. Pathologist Staining for HER2 was graded on a scale of 0–3, with 0 = no reactivity or membranous reactivity in 10% of cells; 1+ = faint/barely perceptible membranous reactivity in 10% of cells or reactivity detected in only part of the cell membrane; 2+ = weak to moderate complete or basolateral membranous reactivity in 10% of tumor cells; and 3+ = strong complete or basolateral membranous reactivity in 10% of tumor cells. In specimens with IHC scores of 2+, DISH (Ventana INFORM HER2 Dual Color ISH, Roche^®^) was also performed. HER2 IHC3+ or IHC2 +/DISH-positive (HER2:CEP17 ratio 2) specimens were defined as HER2-positive. The others were defined as HER2 negative.

### ELISA

We selected the ligands, such as EGF, TGF-alpha, AREG, EREG, NRG, HGF and IGF-1, that had previously been reported to be associated with activation or cross-talk of the downstream signal pathway of the ErbB family in solid tumors. We used ELISA kits to measure serum levels of ligands as follows: Human HGF Quantikine ELISA Kit (DHG00, R & D Systems, Minneapolis, MN, USA), Human epiregulin ELISA kit (CSB-EL007779HU, CUSABIO, Wuhan, Hubei, China), Human amphiregulin ELISA kit (E90006Hu, Uscn Life Science, Wuhan, Hubei, China), Human EGF Quantikine ELISA kit (DEG00, R & D System, Minneapolis, MN, USA), Human TGF-alpha Quantikine ELISA kit (DTGA00, R & D System, Minneapolis, MN, USA), Human Neureglin-1 ELISA kit (CSB-E17153 h, CUSABIO, Wuhan, Hubei, China) and Human IGF-1 Quantikine ELISA kit (DG00, R & D System, Minneapolis, MN, USA). Protocols for ELISA of these ligands are summarized in Supplementary Material 2.

### Assessment and statistical analysis

Assessment of therapeutic response consisted of complete response (CR), partial response (PR), stable disease (SD), disease progression (PD), not evaluated (NE), according to the Response Evaluation Criteria in Solid Tumors (RECIST) criteria ver. 1.1. Response rate (ORR) was defined as the proportion of patients whose best response was a CR or PR among all patients. Disease control rate (DCR) was defined as the proportion of patients whose best response was a CR, a PR, or SD. Response to treatment with trastuzumab was evaluated in all patients who were enrolled in this study.

Prognostic analyses were performed in patients who received fluoropyrimidine plus cisplatin with trastuzumab and paclitaxel plus trastuzumab. Progression-free survival (PFS) was defined as the interval from initiation of trastuzumab to the occurrence of disease progression or death without evidence of progression. Overall survival (OS) was defined as the interval from initiation of trastuzumab to death or last follow-up. Survival curves for PFS and OS were estimated using the Kaplan–Meier method, and differences were evaluated with the log-rank test. Hazard ratios (HRs) and 95% confidence intervals (CIs) were estimated using a Cox proportional hazards model. HRs of univariate analyses were calculated by covariates of performance status (0 vs 1–2), gender (male vs female), age (younger vs older, cut-off; median), primary site (proximal vs distal), stage (stage IV vs recurrence), histological type (intestinal type vs diffuse type), HER2 status (IHC2+/DISH+ vs IHC3+), prior gastrectomy (yes vs no), number of metastatic sites (1 vs 2 or more), peritoneal dissemination (yes vs no), liver metastasis (yes vs no), treatment (fluoropyrimidine plus cisplatin with trastuzumab as first-line chemotherapy vs paclitaxel with trastuzumab as second-line chemotherapy), serum levels of ligands (high vs low, cut-off level: median). HRs of multivariate analyses were calculated by covariates of treatment, serum level of ligands and patient characteristics, which were calculated as *p*-value < 0.1 by univariate analyses.

Differences in the distributions of two variables were evaluated using Fisher exact test or χ^2^ test, as appropriate. Differences in distributions of more than two variables were evaluated by the Kruskal–Wallis test. For continuous variables, differences of medians between two groups were evaluated by the Mann-Whitney *U* test and differences of mean values between two groups were evaluated by the student *t* test in the present study. Receiver operating characteristics (ROC) curve analysis was performed to determine the optimal cut-off values for serum ligands as continuous variables. All tests were two-sided and a *p*-value < 0.05 was defined as statistically significant. We performed statistical analyses by SPSS statistical software, version 19 (IBM, Tokyo, Japan).

## SUPPLEMENTARY MATERIALS



## References

[1] Jemal A, Bray F, Center MM, Ferlay J, Ward E, Forman D (2011). Global cancer statistics. CA: a cancer journal for clinicians.

[2] Katanoda K, Hori M, Matsuda T, Shibata A, Nishino Y, Hattori M, Soda M, Ioka A, Sobue T, Nishimoto H (2015). An updated report on the trends in cancer incidence and mortality in Japan, 1958–2013. Jpn J Clin Oncol.

[3] Popescu NC, King CR, Kraus MH (1989). Localization of the human erbB-2 gene on normal and rearranged chromosomes 17 to bands q12–21. 32. Genomics.

[4] Sheng WQ, Huang D, Ying JM, Lu N, Wu HM, Liu YH, Liu JP, Bu H, Zhou XY, Du X (2013). HER2 status in gastric cancers: a retrospective analysis from four Chinese representative clinical centers and assessment of its prognostic significance. Ann Oncol.

[5] Gravalos C, Jimeno A (2008). HER2 in gastric cancer: a new prognostic factor and a novel therapeutic target. Ann Oncol.

[6] Begnami MD, Fukuda E, Fregnani JH, Nonogaki S, Montagnini AL, da Costa WL, Soares FA (2011). Prognostic implications of altered human epidermal growth factor receptors (HERs) in gastric carcinomas: HER2 and HER3 are predictors of poor outcome. J Clin Oncol.

[7] Qiu MZ, Li Q, Wang ZQ, Liu TS, Liu Q, Wei XL, Jin Y, Wang DS, Ren C, Bai L (2014). HER2-positive patients receiving trastuzumab treatment have a comparable prognosis with HER2-negative advanced gastric cancer patients: a prospective cohort observation. Int J Cancer.

[8] Kim KC, Koh YW, Chang HM, Kim TH, Yook JH, Kim BS, Jang SJ, Park YS (2011). Evaluation of HER2 protein expression in gastric carcinomas: comparative analysis of 1,414 cases of whole-tissue sections and 595 cases of tissue microarrays. Ann Surg Oncol.

[9] Jørgensen JT (2014). Role of human epidermal growth factor receptor 2 in gastric cancer: biological and pharmacological aspects. World J Gastroenterol.

[10] Bang YJ, Van Cutsem E, Feyereislova A, Chung HC, Shen L, Sawaki A, Lordick F, Ohtsu A, Omuro Y, Satoh T (2010). Trastuzumab in combination with chemotherapy versus chemotherapy alone for treatment of HER2-positive advanced gastric or gastro-oesophageal junction cancer (ToGA): a phase 3, open-label, randomised controlled trial. Lancet.

[11] Kurokawa Y, Sugimoto N, Miwa H, Tsuda M, Nishina S, Okuda H, Imamura H, Gamoh M, Sakai D, Shimokawa T, Komatsu Y, Doki Y, Tsujinaka T, Furukawa H (2014). Phase II study of trastuzumab in combination with S-1 plus cisplatin in HER2-positive gastric cancer (HERBIS-1). Br J Cancer.

[12] Iwasa S, Nishikawa K, Miki A, Noshiro H, Tsuburaya A, Nishida Y, Miwa H, Masuishi T, Yoshida K, Kodera Y, Boku N, Yamada Y, Morita S (2013). Multicenter, phase II study of trastuzumab and paclitaxel to treat HER2-positive, metastatic gastric cancer patients naive to trastuzumab (JFMC45–1102). J Clin Oncol.

[13] Bilici A (2014). Treatment options in patients with metastatic gastric cancer: current status and future perspectives. World J Gastroenterol.

[14] Yarden Y (2001). The EGFR family and its ligands in human cancer: signaling mechanisms and therapeutic opportunities. Eur J Cancer.

[15] E Engelman JA, Zejnullahu K, Mitsudomi T, Song Y, Hyland C, Park JO, Lindeman N, Gale CM, Zhao X, Christensen J, Kosaka T, Holmes AJ, Rogers AM (2007). Met amplification leads to gefitinib resistance in lung cancer by activating ERBB3 signaling. Science.

[16] Liska D, Chen CT, Bachleitner-Hofmann T, Christensen JG, Weiser MR (2011). HGF rescues colorectal cancer cells from EGFR inhibition via MET activation. Clin Cancer Res.

[17] Bardelli A, Corso S, Bertotti A, Hobor S, Valtorta E, Siravegna G, Sartore-Bianchi A, Scala E, Cassingena A, Zecchin D, Apicella M, Migliardi G, Galimi F (2013). Amplification of the MET receptor drives resistance to anti-EGFR therapies in colorectal cancer. Cancer Discov.

[18] Agarwal S, Zerillo C, Kolmakova J, Christensen JG, Harris LN, Rimm DL, Digiovanna MP, Stern DF (2009). Association of constitutively activated hepatocyte growth factor receptor (Met) with resistance to a dual EGFR/Her2 inhibitor in non-small-cell lung cancer cells. Br J Cancer.

[19] Liu S (2015). HGF-MET as a breast cancer biomarker.

[20] Cañadas I, Taus A, González I, Villanueva X, Gimeno J, Pijuan L, Dómine M, Sánchez-Font A, Vollmer I, Menéndez S, Arpí O, Mojal S, Rojo F (2014). High circulating hepatocyte growth factor levels associate with epithelial to mesenchymal transition and poor outcome in small cell lung cancer patients. Oncotarget.

[21] Balañá ME, Labriola L, Salatino M, Movsichoff F, Peters G, Charreau EH, Elizalde PV (2001). Activation of ErbB-2 via a hierarchical interaction between ErbB-2 and type I insulin-like growth factor receptor in mammary tumour cells. Oncogene.

[22] Nahta R, Yuan LX, Zhang B, Kobayashi R, Esteva FJ (2005). Insulin-like growth factor-I receptor/human epidermal growth factor receptor 2 heterodimerization contributes to trastuzumab resistance of breast cancer cells. Cancer Res.

[23] Browne BC, Eustace AJ, Kennedy S, O'Brien NA, Pedersen K, McDermott MS, Larkin A, Ballot J, Mahgoub T, Sclafani F, Madden S, Kennedy J, Duffy MJ (2012). Evaluation of IGF1R and phosphorylated IGF1R as targets in HER2-positive breast cancer cell lines and tumours. Breast Cancer Res Treat.

[24] Hofmann M, Stoss O, Shi D, Büttner R, van de Vijver M, Kim W, Ochiai A, Rüschoff J, Henkel T (2008). Assessment of a HER2 scoring system for gastric cancer: results from a validation study. Histopathology.

[25] Chen FL, Xia W, Spector NL (2008). Acquired Resistance to Small Molecule ErbB2 Tyrosine Kinase Inhibitors. Clin Cancer Res.

[26] Thery JC, Spano JP, Azria D, Raymond E, Penault Llorca F (2014). Resistance to human epidermal growth factor receptor type 2-targeted therapies. Eur J Cancer.

[27] Hack SP, Bruey JM, Koeppen H (2014). HGF/MET-directed therapeutics in gastroesophageal cancer: a review of clinical and biomarker development. Oncotarget.

[28] Takahashi N, Yamada Y, Furuta K, Honma Y, Iwasa S, Takashima A, Kato K, Hamaguchi T, Shimada Y (2014). Serum levels of hepatocyte growth factor and epiregulin are associated with the prognosis on anti-EGFR antibody treatment in KRAS wild-type metastatic colorectal cancer. Br J Cancer.

[29] Khoury H, Naujokas MA, Zuo D, Sangwan V, Frigault MM, Petkiewicz S, Dankort DL, Muller WJ, Park M (2005). HGF converts ErbB2/Neu epithelial morphogenesis to cell invasion. Mol Biol Cell.

[30] Minuti G, Cappuzzo F, Duchnowska R, Jassem J, Fabi A, O'Brien T, Mendoza AD, Landi L, Biernat W, Czartoryska-Arłukowicz B, Jankowski T, Zuziak D, Zok J (2012). Increased MET and HGF gene copy numbers are associated with trastuzumab failure in HER2-positive metastatic breast cancer. Br J Cancer.

[31] Shattuck DL, Miller JK, Carraway KL, Sweeney C (2008). Met receptor contributes to trastuzumab resistance of Her2-overexpressing breast cancer cells. Cancer Res.

[32] Zhang Z, Wang J, Ji D, Wang C, Liu R, Wu Z, Liu L, Zhu D, Chang J, Geng R, Xiong L, Fang Q, Li J (2014). Functional genetic approach identifies MET, HER3, IGF1R, INSR pathways as determinants of lapatinib unresponsiveness in HER2-positive gastric cancer. Clin Cancer Res.

[33] Chen CT, Kim H, Liska D, Gao S, Christensen JG, Weiser MR (2012). MET activation mediates resistance to lapatinib inhibition of HER2-amplified gastric cancer cells. Mol Cancer Ther.

[34] Park D, Yoon C, Thomas N, Ku G, Janjigian Y, Kelsen D, Ilson D, Goodman K, Tang L, Strong V, Coit D, Yoon S (2014). Prognostic Significance of Targetable Angiogenic and Growth Factors in Patients Undergoing Resection for Gastric and Gastroesophageal Junction Cancers. Ann Surg Oncol.

[35] Lee HE, Kim MA, Lee HS, Jung EJ, Yang HK, Lee BL, Bang YJ, Kim WH (2012). MET in gastric carcinomas: comparison between protein expression and gene copy number and impact on clinical outcome. Br J Cancer.

[36] Kawakami H, Okamoto I, Arao T, Okamoto W, Matsumoto K, Taniguchi H, Kuwata K, Yamaguchi H, Nishio K, Nakagawa K, Yamada Y (2013). MET amplification as a potential therapeutic target in gastric cancer. Oncotarget.

[37] Lennerz JK, Kwak EL, Ackerman A, Michael M, Fox SB, Bergethon K, Lauwers GY, Christensen JG, Wilner KD, Haber DA, Salgia R, Bang Y-J, Clark JW (2011). MET Amplification Identifies a Small and Aggressive Subgroup of Esophagogastric Adenocarcinoma With Evidence of Responsiveness to Crizotinib. J Clin Oncol.

[38] Toiyama Y, Yasuda H, Saigusa S, Matushita K, Fujikawa H, Tanaka K, Mohri Y, Inoue Y, Goel A, Kusunoki M (2012). Co-expression of hepatocyte growth factor and c-Met predicts peritoneal dissemination established by autocrine hepatocyte growth factor/c-Met signaling in gastric cancer. Int J Cancer.

[39] Graziano F, Galluccio N, Lorenzini P, Ruzzo A, Canestrari E, D'Emidio S, Catalano V, Sisti V, Ligorio C, Andreoni F, Rulli E, Di Oto E, Fiorentini G (2011). Genetic Activation of the MET Pathway and Prognosis of Patients With High-Risk, Radically Resected Gastric Cancer. J Clin Oncol.

[40] Nagatsuma AK, Aizawa M, Kuwata T, Doi T, Ohtsu A, Fujii H, Ochiai A (2015). Expression profiles of HER2, EGFR, MET and FGFR2 in a large cohort of patients with gastric adenocarcinoma. Gastric Cancer.

[41] Ha SY, Lee J, Jang J, Hong JY, Do IG, Park SH, Park JO, Choi MG, Sohn TS, Bae JM, Kim S, Kim M, Kim S (2015). HER2-positive gastric cancer with concomitant MET and/or EGFR overexpression: a distinct subset of patients for dual inhibition therapy. Int J Cancer.

[42] Janjigian YY, Werner D, Pauligk C, Steinmetz K, Kelsen DP, Jäger E, Altmannsberger HM, Robinson E, Tafe LJ, Tang LH, Shah MA, Al-Batran SE (2012). Prognosis of metastatic gastric and gastroesophageal junction cancer by HER2 status: a European and USA International collaborative analysis. Ann Oncol.

[43] Matsubara J, Yamada Y, Hirashima Y, Takahari D, Okita NT, Kato K, Hamaguchi T, Shirao K, Shimada Y, Shimoda T (2008). Impact of insulin-like growth factor type 1 receptor, epidermal growth factor receptor, and HER2 expressions on outcomes of patients with gastric cancer. Clin Cancer Res.

[44] Honma Y, Shimada Y, Takashima A, Iwasa S, Kato K, Hamaguchi T, Yamada Y, Taniguchi H, Sekine S, Kushima R (2014). Efficacy of S-1 plus cisplatin combination chemotherapy in patients with HER2-positive advanced gastric cancer. Int J Clin Oncol.

[45] Iveson T, Donehower RC, Davidenko I, Tjulandin S, Deptala A, Harrison M, Nirni S, Lakshmaiah K, Thomas A, Jiang Y, Zhu M, Tang R, Anderson A (2014). Rilotumumab in combination with epirubicin, cisplatin, and capecitabine as first-line treatment for gastric or oesophagogastric junction adenocarcinoma: an open-label, dose de-escalation phase 1b study and a double-blind, randomised phase 2 study. Lancet Oncol.

[46] Cunningham D, Tebbutt NC, Davidenko I, Murad AM, Al-Batran SE, Ilson DH, Tjulandin S, Gotovkin E, Karaszewska B, Bondarenko I, Tejani MA, Udrea AA, Tehfe MA (2015). Phase III, randomized, double-blind, multicenter, placebo (P)-controlled trial of rilotumumab (R) plus epirubicin, cisplatin and capecitabine (ECX) as first-line therapy in patients (pts) with advanced MET-positive (pos) gastric or gastroesophageal junction (G/GEJ) cancer: RILOMET-1 study. J Clin Oncol.

[47] Shah MA, Bang YJ, Lordick F, Tabernero J, Chen M, Hack SP, Phan SC, Shames DS, Cunningham D (2015). METGastric: A phase III study of onartuzumab plus mFOLFOX6 in patients with metastatic HER2-negative (HER2-) and MET-positive (MET+) adenocarcinoma of the stomach or gastroesophageal junction (GEC). J Clin Oncol.

[48] Kondratov RV, Kondratova AA (2014). Rapamycin in preventive (very low) doses. Aging (Albany NY).

