# Supplementary material for: Serum level of hepatocyte growth factor is a novel marker of predicting the outcome and resistance to the treatment with trastuzumab in HER2-positive patients with metastatic gastric cancer
Source: Oncotarget. 2015 Dec 24;7(4):4925–38. doi: 10.18632/oncotarget.6753 (PMC4826254; doi:10.18632/oncotarget.6753)
Supplement: Supplementary file 1 [file oncotarget-07-4925-s001.pdf]

Serum level of hepatocyte growth factor is a novel marker of predicting the outcome and resistance to the treatment with trastuzumab in HER2-positive patients with metastatic gastric cancer

Supplementary Materials

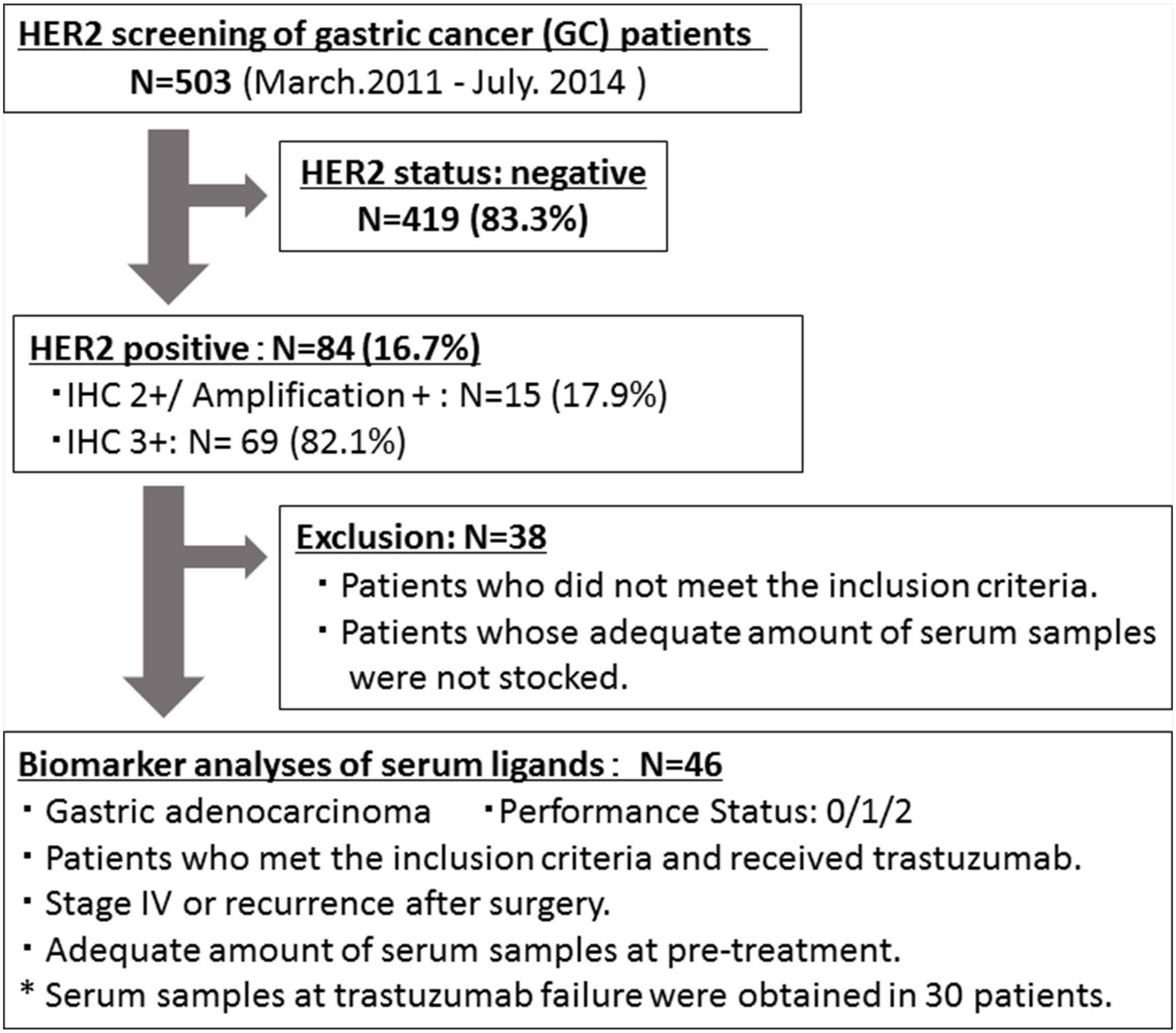

Supplementary Data S1: Diagrame of this study.

Supplementary Material S2: Protocols of ELISA of serum ligands

ELISA-HGF

(1) we prepared all reagents, standard dilutions, and samples as directed in the product insert, (2) added 150 µL of assay diluent to each well and then 50 µL of standard and sample to each well, (3) covered with a plate sealer and incubated at room temperature for 2 hours, (4) aspirated each well and washed, repeating the process 3 times for a total of 4 washes, (5) we added 200 µL of HRP conjugate to each well, (6) covered with a new plate sealer, incubated at room temperature for 2 hours, and aspirated and washed 4 times, (7) we added 200 µL TMB substrate solution to each well and incubated at room temperature for 30 minutes with protection from light, (8) we added 50 µL of stop solution to each well, (9) read at 450 nm within 30 minutes and set wavelength correction to 540 nm or 570 nm.

ELISA-Epiregulin

(1) we prepared all reagents, standards, and samples (5x diluted with sample diluent), (2) added 100 µl of standard and sample per well and covered with a plate sealer, and then incubated for 2 hours at 37°C, (3) removed the liquid from each well, (4) added 100 µl of Biotin-antibody (1x) to each well and covered with a plate sealer, and then incubated for 1 hour at 37°C, (5) aspirated each well and washed, repeating the process two times for a total of three washes, (6) added 100 µl of HRP-avidin (1x) to each well and covered with a plate sealer, and incubated for 1 hour at 37°C, (7) repeated the aspiration/wash process five times, (8) added 90 µl of TMB substrate to each well and incubated for 15–30 minutes at 37°C with protection from light, (9) added 50 µl of Stop Solution to each well, read at 450 nm within 30 minutes and set wavelength correction to 540 nm or 570 nm.

ELISA-Amphiregulin

(1) we prepared all reagents, standards, and samples, (2) added 100 µl of standard and sample per well and covered with a plate sealer, and then incubated for 2 hours at 37°C, (3) removed the liquid from each well, (4) Added 100 µL of Detection Reagent A working solution to each well, and then incubated for 1 hour at 37°C, (5) added 350 µL of washed buffer and aspirate each well and wash, repeating the process twice for a total of three washes, (6) added 100 µL of Detection Reagent B working solution to each well and incubated for 30 minutes at 37°C after covering it with the Plate sealer, (7) repeated the aspiration/wash process three times, (8) added 90 µl of TMB substrate to each well and incubated for 25–30 minutes at 37°C with protection from light, (9) added 50 µl of Stop Solution to each well, read at 450 nm within 30 minutes.

ELISA-EGF

(1) we prepared all reagents, standards and samples, and then 50 µL Assay Diluent for serum samples, (2) added 200 µl of standard and sample per well and covered with a plate sealer, and then incubated for 2 hours at 37°C, (3) aspirated each well and wash, repeating the process twice for a total of 3 washes, (4) added 200 µL of Conjugate to each well and covered with a new plate sealer, and then incubated at room temperature for 2 hours, (5) aspirated each well and wash, repeating the process for a total of three washes, (6) added 200 µl of substrate solution to each well and incubated for 20 minutes at 37°C with protection from light, (9) added 50 µl of Stop Solution to each well, read at 450 nm within 30 minutes and set wavelength correction to 540 nm or 570 nm.

ELISA-TGF alpha

(1) we prepared all reagents, standards, and samples, (2) added 50 µl of standard and sample per well and covered with a plate sealer, and then incubated for 2 hours at 37°C, (3) aspirated each well and wash, repeating the process three times for a total of 4 washes (4) added 200 µL of Conjugate to each well, and covered with a new plate sealer, and incubate at room temperature for 2 hours, (5) aspirated each well and washed four times, (6) added 200 µl of substrate solution to each well and incubated for 30 minutes at 37°C with protection from light, (9) added 50µl of Stop Solution to each well, read at 450 nm within 30 minutes and set wavelength correction to 540 nm or 570 nm.

ELISA-Neureglin 1

(1) we prepared all reagents, standards, and samples, (2) added 100 µl of standard and sample per well and covered with a plate sealer, and then incubated for 2 hours at 37°C, (3) removed the liquid from each well, (4) added100 µl of Biotin-antibody (1x) to each well and covered with a new adhesive strip, and then incubated for 1 hour at 37°C, (5) aspirate each well and wash, repeating the process two times for a total of three washes, (6) added 100 µl of HRP-avidin (1x) to each well and covered the microtiter plate with a new adhesive strip, and then incubated for 1 hour at 37°C, (7) repeated the aspiration/wash process five times, (8) added 90 µl of TMB substrate to each well and incubated for 15–30 minutes at 37°C with protection from light, (9) added 50 µl of Stop Solution to each well, read at 450 nm within 30 minutes and set wavelength correction to 540 nm or 570 nm.

ELISA-IGF-1

(1) we prepared all reagents, standards, and samples, (2) added 150 μL of Assay Diluent to each well and added 50 μl of standard and sample per well, and then covered with a plate sealer and incubated for 2 hours at 4°C, (3) aspirated each well and washed, repeating the process 3 times for a total of four washes, (4) Added 200 μL of cold Conjugate to each well and covered with a new plate sealer, and incubated at 4°C for 1 hour, (5) aspirated each well and wash four times, (8) added 200 μl of TMB substrate to each well and incubated for 30 minutes at 37°C with protection from light, (9) added 50 μl of Stop Solution to each well, read at 450 nm within 30 minutes and set wavelength correction to 540 nm or 570 nm.

Supplementary Material S3: Comparison of patient's characteristics by serum levels (high/low) of HGF and EREG (cut-off value: median)

	HGF low (*n* = 23)	HGF high (*n* = 23)	*p*-value
**Median Age (range)**	68 (36–85)	67.5 (45–80)	
**Gender (%)**			1.000
Male	21 (91.3)	22 (95.7)	
Female	2 (8.7)	1 (4.3)	
**ECOG PS (%)**			1.000
0–1	22 (95.7)	22 (95.7)	
2	1 (4.3)	1 (4.3)	
**Histological type (%)**			0.514
Differentiated	18 (78.3)	15 (65.2)	
Undifferentiated	5 (21.7)	8 (34.8)	
**Primary site (%)**			0.768
Proximal	12 (52.2)	10 (43.5)	
Distal	11 (47.8)	13 (56.5)	
**Number of metastatic site (%)**			0.376
1	13 (56.5)	9 (39.1)	
2 ≤	10 (43.5)	14 (60.9)	
**Metastatic site (%)**			
Liver	10 (43.5)	17 (73.9)	0.071
Lung	3 (13.0)	1 (4.3)	0.608
Peritoneum	5 (21.7)	8 (34.8)	0.514
Lymph node	18 (78.3)	15 (65.2)	0.514
**HER2 status (%)**			1.000
IHC 3+	19 (82.6)	20 (87.0)	
IHC 2+ / amplification +	4 (17.4)	3 (13.0)	

	EREG low (*n* = 23)	EREG high (*n* = 23)	*p*-value
**Median Age (range)**	68.0 (41–85)	67.0 (36–82)	
**Gender (%)**			1.000
Male	21 (91.3)	22 (95.7)	
Female	2 (8.7)	1 (4.3)	
**ECOG PS (%)**			1.000
0–1	22 (95.7)	23 (95.7)	
2	1 (4.3)	1 (4.3)	
**Histological type (%)**			0.514
Differentiated	18 (78.3)	15 (65.2)	
Undifferentiated	5 (21.7)	8 (34.8)	
**Primary site (%)**			1.000

Proximal	11 (47.8)	11 (47.8)	
Distal	12 (52.2)	12 (52.2)	
**Number of metastatic site (%)**			1.000
1	11 (47.8)	10 (43.5)	
2 ≤	12 (52.2)	13 (56.5)	
**Metastatic site (%)**			
Liver	13 (56.5)	14 (60.9)	1.000
Lung	4 (17.4)	2 (8.7)	0.665
Peritoneum	3 (13.0)	10 (43.5)	0.047
Lymph node	18 (78.3)	15 (65.2)	0.514
**HER2 status (%)**			1.000
IHC 3+	19 (82.6)	20 (87.0)	
IHC 2+ / amplification +	4 (17.4)	3 (13.0)	

Abbreviations: ECOG = Eastern Cooperative Oncology Group, PS = performance status, IHC = immunohistochemistry.

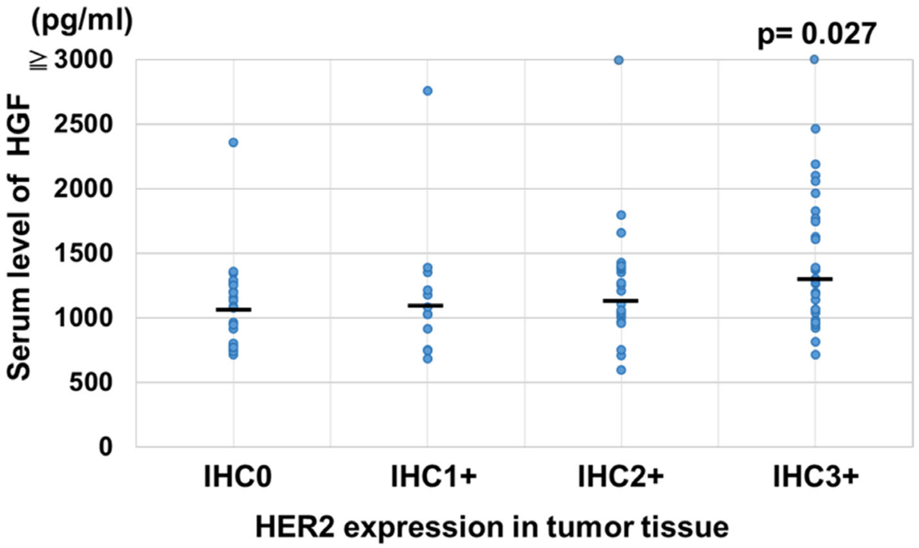

Supplementary Data S4: Serum levels of HGF by HER2 expression in HER2- positive gastric canceer patients who received first-line chemotherapy with trastuzumab.
